# SIRT4 Is Highly Expressed in Retinal Müller Glial Cells

**DOI:** 10.3389/fnins.2022.840443

**Published:** 2022-02-04

**Authors:** Wei Wei, Piaopiao Hu, Mengqi Qin, Guiping Chen, Feifei Wang, Shengrui Yao, Ming Jin, Zhi Xie, Xu Zhang

**Affiliations:** Jiangxi Provincial Key Laboratory for Ophthalmology, Jiangxi Clinical Research Center of Ophthalmic Disease, Affiliated Eye Hospital of Nanchang University, Nanchang, China

**Keywords:** SIRT4, Müller glia cell, glutamine synthetase, resveratrol, retina, excitotoxicity

## Abstract

Sirtuin 4 (SIRT4) is one of seven mammalian sirtuins that possesses ADP-ribosyltransferase, lipoamidase and deacylase activities and plays indispensable role in metabolic regulation. However, the role of SIRT4 in the retina is not clearly understood. The purpose of this study was to explore the location and function of SIRT4 in the retina. Therefore, immunofluorescence was used to analyze the localization of SIRT4 in rat, mouse and human retinas. Western blotting was used to assess SIRT4 and glutamine synthetase (GS) protein expression at different developmental stages in C57BL/6 mice retinas. We further analyzed the retinal structure, electrophysiological function and the expression of GS protein in SIRT4-deficient mice. Excitotoxicity was caused by intravitreal injection of glutamate (50 nmol) in mice with long-term intraperitoneal injection of resveratrol (20 mg/Kg), and then retinas were subjected to Western blotting and paraffin section staining to analyze the effect of SIRT4 on excitotoxicity. We show that SIRT4 co-locates with Müller glial cell markers (GS and vimentin). The protein expression pattern of SIRT4 was similar to that of GS, and both increased with development. There were no significant retinal structure or electrophysiological function changes in 2-month SIRT4-deficient mice, while the expression of GS protein was decreased. Moreover, long-term administration of resveratrol can upregulate the expression of SIRT4 and GS while reducing the retinal injury caused by excessive glutamate. These results suggest that SIRT4 is highly expressed in retinal Müller glial cells and is relevant to the expression of GS. SIRT4 does not appear to be essential in retinal development, but resveratrol, as an activator of SIRT4, can upregulate GS protein expression and protect the retina from excitotoxicity.

## Introduction

Sirtuins are a class of histone deacetylases that regulate a range of pathophysiological processes, including cell senescence, inflammation, metabolism, and cell proliferation (Luo et al., 2017; Yang et al., 2020). Mammals have seven kinds of sirtuins (SIRT1-7), among them, SIRT3-5 is expressed in cell mitochondria. Previous studies have explored the role of SIRT3 and SIRT5 in the retina (Lin et al., 2016), while few studies have investigated the role of SIRT4 in the retina. SIRT4 is one of seven mammalian sirtuins that use NAD to ADP-ribosylate and downregulate glutamate dehydrogenase (GDH) activity. SIRT4 inhibits the enzymatic activity of GDH and limits the metabolism of glutamate and glutamine in order to produce ATP (Haigis et al., 2006). The physiological effects of SIRT4 include the role of the insulin response (Haigis et al., 2006), fatty acid metabolism (Laurent et al., 2013), tumor inhibition (Jeong et al., 2013), and regulation of ATP levels in muscles and liver (Ho et al., 2013). In the brain, SIRT4 is localized to mitochondria, expressed at high levels in astrocytes in the postnatal brain and radial glia in embryonic tissues, and shows a lower expression during development (Komlos et al., 2013). However, little is known about the function of SIRT4 in the retina, especially its role in the development of Müller glial cells (MGCs). Recently, there have been new insights into the activity of SIRT4 that SIRT4 is a lysine deacylase (Anderson et al., 2017). Our understanding of SIRT4 enzyme activity is still incomplete and whether there are other enzyme activities of SIRT4 in MGCs remains to be explored.

MGCs have a unique localization, in that they span the entire retina and are present between vessels and neurons (Bringmann et al., 2006; Goldman, 2014; Toft-Kehler et al., 2017). Loss of mature MGCs in a range of species leads to disruptions in retinal structure, which causes impaired neuronal function and visual deficits (Wohl et al., 2017). MGC dysfunction is implicated in many retinal diseases, such as glaucoma, proliferative retinopathies and age-related macular degeneration (AMD) (Devoldere et al., 2019; Simón et al., 2019). MGCs participate in the uptake glutamate released by synapses and amidate glutamate to the nontoxic amino acid glutamine by glutamine synthetase (GS) (Fletcher et al., 2005). Numerous studies have demonstrated that MGC dysfunction always results in an increase in retinal glutamate level that leads to excitatory neural toxicity (Bringmann et al., 2009; Mysona et al., 2009; Bringmann and Wiedemann, 2011). Our previous studies suggest that SIRT4 protein is the only filamentous expression of the sirtuins family in the retina of adult rats (Luo et al., 2017). The expression pattern of SIRT4 is similar to that of MGCs, suggesting that SIRT4 is associated with the function of MGCs.

Resveratrol (RES) is a kind of SIRT4 activator that increases SIRT4 gene and protein expression (Sheng et al., 2018). In this study, we upregulated SIRT4 by intraperitoneal injection of RES to explore the influence of SIRT4 on the retina. Given that SIRT4 is a therapeutic target for neurodegenerative disorders (Dai et al., 2018), and plays a role in the metabolism of glutamate and glutamine (Haigis et al., 2006), we wanted to determine whether SIRT4 works in excitatory toxicity caused by glutamate and the role of GS. Although SIRT4 protein and MGCs have similar localization in the retina, and both participate in the regulation of glutamate-glutamine, there are still many questions. Whether SIRT4 affects the morphology and function of MGCs and even the retina remains unknown.

Thus, we studied the location of SIRT4 in the retina and explore whether SIRT4 affects retinal structure and function by regulating MGC maturation to clarify the role of SIRT4 in the retina. Furthermore, SIRT4 expression was downregulated by gene knockout and upregulated by intraperitoneal injection of RES to study the effects of SIRT4 on GS protein expression and retinal structure.

## Materials and Methods

### Tissue Preparation

C57BL/6 mice and Sprague-Dawley (SD) rats aged 6-8 weeks were originally obtained from Hunan Slac Jinda Laboratory Animal Co., Ltd. (license number: SCXK1201-0004) and the Center of Jiangxi University of Traditional Chinese Medicine (license number: SCXX12018-0003), respectively. SIRT4 knockout (KO) mice from a pure C57BL/6 background were produced by Cyagen Biosciences, Suzhou, China. Mice were interbred to yield SIRT4*^KO^* mice and the corresponding wide-type control. Genotypes were determined by PCR analysis from genomic DNA obtained from tail biopsy specimens before the experiments. The animals were housed under standard conditions with free access to food and water and were maintained in temperature-controlled rooms on a normal 12-h light/12-h dark cycle. All experiments were conducted in accordance with the Animal Care and Use Committees of Nanchang University Medical School.

Three pairs of donor eyes of different ages (age range 18-59 years old) were obtained from the Red Cross Society of China Jiangxi Branch, and all volunteers signed informed consent documents in written form in accordance with the principles of the Declaration of Helsinki. All experiments involving human samples were approved by the Ethics Committee of Affiliated Eye Hospital of Nanchang University. To obtain fresh eye tissues for protein extraction and staining, we restricted the samples in the present study to those received less than 48 h postmortem. The previous medical and ocular histories of all donors were assessed to exclude donors with any eye disease. The retinas were processed as described below.

### Drug Treatment

Resveratrol (RES) (3,4′,5-trihydroxy-trans-stilbene, Sigma-Aldrich) was dissolved in 6.67% dimethylsulfoxide (DMSO) to a concentration of 5 mg/ml. Two-month-old C57BL/6 mice were intraperitoneally injected with RES (20 mg/kg) for five consecutive days before glutamate intravitreal injection. The control group was intraperitoneally injected with an equal volume of 6.67% DMSO.

### Intravitreal Injection

The mice were anesthetized by i.p., injection of 3.6% chloral hydrate (10 ml/kg). Before injection, pupillary dilatation was maintained by administering tropicamide phenylephrine eye drops 3 times (Santen Pharmaceutical Co., Ltd., Japan), and topical anesthesia was achieved by administering proparacaine hydrochloride eye drops 3 times (Alcon Co., Ltd., Belgium). Levofloxacin eye drops (Santen Pharmaceutical Co., Ltd., Japan) were administered three times to clean conjunctival sacs.

One microliter 50 nmol glutamate (Solarbio) soluble in 37°C normal saline (NS) was injected into the right eye with a 30-gauge (30 G) needle (Hamilton). The needle punctured the center of the vitreous chamber approximately 1.5 mm from the limbus in the superior temporal quadrant. The tip of the needle was pointed in the direction of the optic nerve to avoid injuring the lens. The tip was visible through the dilated pupil, and the drug was slowly injected. The needle was left in the eye for an additional 20 s to allow the eye to adjust to the increase in volume and then pulled out. The left eye received the same volume of NS as this was used as the solvent for glutamate. After injection, the eyes were treated three times with levofloxacin eye drops (Santen Pharmaceutical Co., Ltd., Japan). While recovering from anesthesia, the animals were placed in their cages. At least three animals were used for each experimental condition.

### Histopathology

The eyes were enucleated and fixed in FAS for 24 h at 4°C. After washing in PBS, the tissues were transferred to 70% ethanol overnight, then dehydrated and embedded in paraffin. Fixed eyeballs were cut into 5-μm-thick pieces parallel to the maximal circumference of the eyeball through the optic disk. The tissues were stained with hematoxylin and eosin (H&E) and observed under light microscope (Leica, Heidelberg, Germany). Light microscope images were obtained at 20 × and 40 × magnification. To evaluate the differences in retinal structure, the thickness of the total retina, the ganglion cell layer (GCL), the inner plexiform layer (IPL), the inner nuclear layer (INL), the outer plexiform layer (OPL), the outer nuclear layers (ONL) and the layer of photoreceptor outer segment (POSL). All measurements were measured at 1 mm away from the center of the optic disk, and three sections per eye were averaged.

### TUNEL Assay

For the detection of apoptotic cells, terminal deoxynucleotidyl transferase dUTP nick end labeling (TUNEL) staining was performed using the TransDetect *In situ* Fluorescein TUNEL Cell Apoptosis Detection Kit (TransGen Biotech, Beijing, China). Before staining, the paraffin sections were dewaxed and rehydrated. The nuclei were costained with 4-6-diamidino-2-phenylindole (DAPI, Boster, Wuhan, China).

### Immunofluorescence Staining

The animals were anesthetized with an intraperitoneal injection of 3.6% chloral hydrate (10 ml/kg). Retinas were harvested at postnatal days 5 (P5), 10 (P10), 15 (P15), 25 (P25), and 60 (P60). The eye were fixed in 4% PFA in phosphate-buffered saline (PBS) overnight and sequentially immersed in 10%, 20%, and 30% sucrose in PBS, mounted in optimal cutting temperature (OCT) compound (Tissue-Tek; Sakura Finetek, Torrance, CA, United States) and frozen at −80°C. Frozen sections of eyes were dried at room temperature after cutting to 7 μm thickness with a Leica cryostat (CM1950, Heidelberger, Germany). The sections were washed with PBS and blocked in PBS containing 0.1% Triton X-100 and donkey serum (Solarbio, Beijing, China) for 1 h at room temperature. The slides were incubated with antibody overnight at 4°C. The working dilutions and sources of antibodies are listed in Table 1. The secondary antibodies included donkey-anti-rabbit-AlexaFlour^®^ 488/594, donkey-anti-mouse- AlexaFlour^®^ 488/594, and donkey-anti-goat-AlexaFlour^®^ 488(Abcam, Cambridge, MA, United States) diluted to 1:200 in PBS plus 0.2% Triton X-100 at room temperature for 1 h. Subsequently, the sections were counterstained with DAPI. Coverslips were affixed to glass slides using anti-fading buffer (Bioworld Technology Inc., St. Louis Park, MN, United States) and visually examined under a Zeiss microscope (ZEISS, LSM800, GÖttingen, Germany) equipped with epifluorescence. Digitized images were obtained by using a Zeiss camera and the images were processed and compiled using Photoshop Software. The staining was repeated three or more times for each antibody.

**TABLE 1:** Primary antibodies used in the study.

Antibody	Source	RRID	Type of Antibody	Dilution	MW
SIRT4	Abcam	ab10140	Goat polyclonal	1:100 (IHC) 1:1000 (WB)	36kDa
SIRT4	Abcam	ab124521	Rabbit polyclonal	1:1000 (WB)	32kDa
GS	Abcam	ab64613	Mouse mAb	1:100 (IHC)	37kDa
GS	Abcam	ab49873	Rabbit polyclonal	1:1000 (WB)	42kDa
Vimentin	CST	#5741	Rabbit mAb	1:100 (IHC) 1:1000 (WB)	57kDa
GFAP	Sigma	G3893	Mouse mAb	1:100 (IHC)	50kDa
CRALBP	Abcam	Ab243664	Rabbit mAb	1:1000 (WB)	36kDa
β-tubulin	TRANKS	J10715	Donkey anti-mouse	1:1000 (WB)	55kDa
β-actin	SCB	#L0117	Mouse mAb	1:1000 (WB)	42kDa

*RRID = Research Resource Identification number; W = Molecular weight; IHC = immunohistochemical; WB = Western blot; kDa = kilo Dalton.*

### Western Blotting

Retinas were lysed in the radioimmune precipitation assay buffer (RIPA) containing PMSF (Solarbio, Beijing, China). The lysates were processed with ultrasound and centrifuged. The protein concentration was determined using the BCA assay (Solarbio, Beijing, China) according to the manufacturers’ instructions. For the Western blot analysis, aliquots containing an equal amount of protein (10 μg) were analyzed by SDS-polyacrylamide gel electrophoresis on 10% gels and were transferred to polyvinylidene fluoride membranes (PVDF, Millipore, United Kingdom), Membranes were blocked with 5% nonfat milk powder dissolved in Tris-buffered saline (TBS) containing 0.1% Tween 20 (TBST) for 1 h and incubated with the appropriate primary antibody (Table 1) overnight at 4°C. Membranes were then washed in TBST and incubated with HRP-conjugated secondary antibodies (Cell Signaling) for 1 h at room temperature. After further washing in TBST, target proteins on the membranes were detected with the EasySee Western Blot Kit (TRANS, Beijing, China) to collect digital images. The bands were quantified by integration of pixel intensity using ImageJ software and normalized to β-tubulin, which served as an internal control.

### Electroretinogram

The mice were subjected to dim red light following overnight dark adaptation ( > 12 h), and anesthetized with isoflurane to induce and chloral hydrate at 3.6% (10 ml/kg) to maintain anesthesia. Then, 1% tropicamide was used to dilate the pupils. The mouse temperature was maintained at 37°C with a heating pad. Stainless steel wire loops (0.1 mm diameter) were placed on the center of the cornea in 1% methylcellulose to prevent corneal dehydration. A reference electrode was placed at the midpoint of the line between the eye and ear and a grounding electrode was placed near the tail. Two-month mice (*n* = 3) were subjected to the guidelines of the International Society for Clinical Electrophysiology of Vision (ISCEV), including the scotopic 0.01 ERG test (rod response) elicited by white light flashes at an intensity of 0.003 phot cd s/m^2^, the scotopic 3.0 ERG test (cone and rod response) elicited by white light flashes at an intensity of 3.0 phot cd s/m^2^, the scotopic 3.0 OPS test (oscillatory potentials) simultaneously elicited by white light flashes at an intensity of 20.0 phot cd s/m^2^, the photopic 3.0 ERG test (cone response) under a white background elicited by white light flashes at an intensity of 2.8 phot cd s/m^2^ and under a white background light at 29.0 phot cd/m^2^ after 10 min of light adaptation. The amplitude of the a-wave was measured from the baseline to the trough, while that of the b-wave was measured from the maximum of the a-wave trough to the peak of the b-wave.

### DNA Extraction and Quantitative PCR Assay

Quantitative PCR was used to measure SIRT4 gene expression in SIRT4*^KO^* mice. Total DNA was extracted from mouse tails with the Ezup column animal genomic DNA extraction kit (B518251; Sangon Biotech, Shanghai, China). Mice were PCR-genotyped using the following primers: Primer 1 (for wide-type allele): 5′- ACGCTACCAACCTAATGGCATC -3′ (forward) and 5′- TCCAGACACCTTGAGTCGCCTAG -3′ (reverse). Primer 2 (for knockout allele): 5′- ACGCTACCAACCTAATGGCATC -3′ (forward) and 5′- GAAGGCGACACAGCTACTCCATC -3′ (reverse). The primers were chosen to amplify DNA using 2xSpecificTMTaq Master Mix (E010; Novoprotein Scientific Inc.). The procedure was initial denaturation (94°C for 3 min), then 35 cycles (94°C for 30 s, 60°C for 35 s and 72°C for 35 s), and then an extension step (72°C for 5 min). All data were chosen from the linear phase of amplification. Amplified DNA was analyzed on a 0.8% agarose gel (with ethidium bromide) and observed by Image Lab under UV light. The DNA Ladder (DM033; Novoprotein Scientific Inc.) were used to benchmark the DNA band sizes.

### Data Analysis

All quantified data represent an average of at least three samples. SPSS 22.0, ImageJ and GraphPad Prism 8.0 software were used for statistical analysis. All data were presented as the mean ± standard error of the mean (SEM). Unpaired Student’s *t*-test was used for two groups of samples to determine the significance of the response, while one-way ANOVA and Post Hoc Turkey Test were used for three or more groups of samples. *P* < 0.05 was considered to be statistically significant.

## Results

### Sirtuin 4 Co-located With GS and Vimentin

**FIGURE 1:**
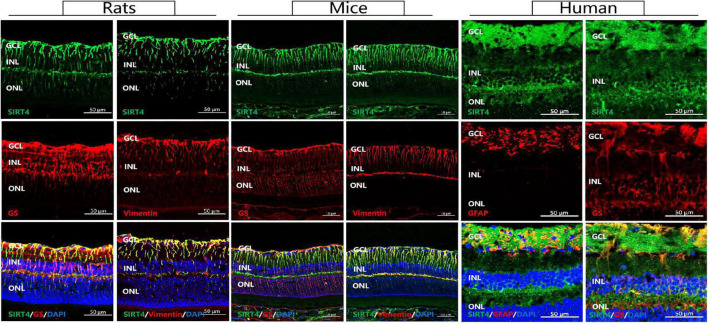
Sirtuin 4 co-localized with MGC markers GS and vimentin. In rats and mice, SIRT4 was mainly expressed in the GCL and the INL in the form of longitudinal filamentous. GCL, ganglion cell layer; INL, inner nuclear layer; ONL, outer nuclear layer. GS, glutamine synthetase; GFAP, glial fibrillary acidic portein; DAPI was used to stain the nuclei (blue). Scale bar = 50 μm.

MGCs span all cellular and plexiform layers of the retina, forming microvilli at the apical surface. We found that in rat, mouse and human retinas SIRT4-positive cells continuously penetrated the inner nuclear layer (INL) and the outer nuclear layer (ONL) in a longitudinal filamentous pattern, consistent with radial glial-like cell localization. To prove that SIRT4 is expressed in MGCs, we used two MGC markers (GS and vimentin) and glial fibrillary acidic protein (GFAP) to costain SIRT4 (Figure 1). Immunostaining showed that SIRT4 co-localized with GS, vimentin and GFAP which means that SIRT4 was highly expressed in MGCs. Notably, SIRT4 was also expressed in the inner plexiform layer (IPL).

### Expression of Sirtuin 4 in the Mice Retinas at Different Developmental Stages

**FIGURE 2:**
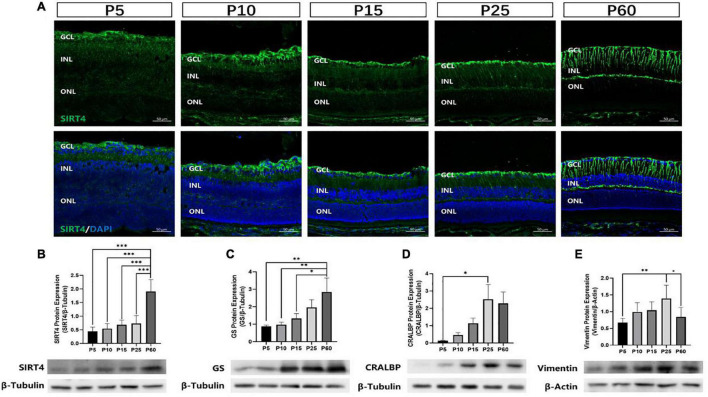
Expression of SIRT4 in mice retinas at different developmental stages. **(A)** Expression pattern of SIRT4 during retinal development in mice retinas by immunohistochemistry at postnatal day 5 (P5), P10, P15, P25, and P60. **(B–E)** Western blot analysis of SIRT4, GS, CRALBP and vimentin protein levels in the retina of C57BL/6 mice at different developmental stages. GCL: ganglion cell layer; INL: inner nuclear layer; ONL: outer nuclear layer. GS: glutamine synthetase; CRALBP: cellular retinaldehyde binding protein; DAPI was used to stain the nuclei (blue). Data are shown as mean ± SEM. (*n* = 6 per group, *P < 0.05, ^**^P < 0.01, ^***^P < 0.001). Scale bar = 50 μm.

To investigate the postnatal expression of SIRT4, we performed immunofluorescence labeling for SIRT4 from postnatal day 5 (P5), 10 (P10), 15 (P15), 25 (P25), and 60 (P60) in the mouse retina (Figure 2A). From P5 to P25, we found that SIRT4 had high expression in the nerve fiber layer (NFL), and SIRT4-positive cell processes ran perpendicular through the layers from the NFL to the ONL. For the P60 groups, the filamentous expression of SIRT4 was intermittently expressed in the inner plexiform layer (IPL), while little expression was noted in the outer plexiform layer (OPL). The protein expression of SIRT4 was detected, and it was found that the expression of SIRT4 increased during retinal development and reached its peak at 2 months (Figure 2B). The growth trend of GS protein expression was similar to that of SIRT4, and also reached its peak at P60, although the protein expression contents of the two were not completely consistent (Figure 2C). Cellular retinaldehyde-binding protein (CRALBP) and vimentin protein expression peaked at P25 (Figures 2D,E).

Retinal progenitor cells (RPCs) give rise to all six types of retinal neurons in a distinct spatiotemporal order spanning embryonic day 12 to postnatal day 10 in the murine retina (Turner and Cepko, 1987). MGCs, which mature later than most retinal cells, were observed to have the lowest GS and SIRT4 protein expression at P5 and the highest at P60. These results indicate that SIRT4 may be a relevant protein in the development of MGCs.

### Retinal Structure of 2-Month Sirtuin 4-Deficient Mice

**FIGURE 3:**
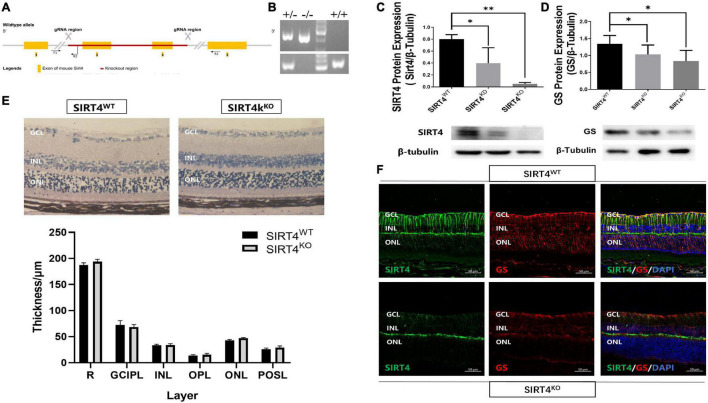
Retinal structure of 2-month SIRT4-deficient mice. **(A)** Schematic diagram of SIRT4 gene knockout. **(B)** Genotyping identification. **(C,D)** SIRT4 and GS protein expression in SIRT4^WT^, SIRT4^KD^ and SIRT4^KO^ mice were analyzed using western blotting. **(E)** Retinal thickness analysis after H&E staining of paraffin sections of SIRT4^WT^ and SIRT4^KO^ mice. **(F)** Immunofluorescence analysis of SIRT4 and GS in the retina of SIRT4^WT^ and SIRT4^KO^ mice. WT, wild type; KD, knock down; KO, knock out; NFL, nerve fiber layer; GCL, ganglion cell layer; GCIPL, ganglion cell-inner plexiform layer; INL, inner nuclear layer; OPL, out plexiform layer; ONL, outer nuclear layer; POSL, photoreceptor outer segment layer; GS, glutamine synthetase; DAPI was used to stain the nuclei (blue). Data are shown as mean ± SEM. (*n* = 3 per group, *P < 0.05, ^**^P < 0.01). Scale bar = 50 μm.

The SIRT4^KO^ mice generated from Cyagen US inc., by removing exons 3-4 of the SIRT4 gene (Figure 3A). PCR was used to identify SIRT4^KO^ mice, SIRT4 knockdown (SIRT4^KD^) mice and wild-type (SIRT4^WT^) mice (Figure 3B). The Western blotting results showed that SIRT4 and GS expression were lower in SIRT4 ^KD^ mice than that in SIRT4^WT^ mice, and SIRT4 was barely expressed in SIRT4^KO^ mice (Figures 3C,D). There was no significant difference in retinal structure between SIRT4^KO^ mice and SIRT4^WT^ mice according to the paraffin section H&E staining analysis (Figure 3E). However, compared with SIRT4^WT^, SIRT4 immunofluorescence, especially filamentous staining in the GCL and the INL, was significantly reduced in SIRT4^KO^. Although the brightness of SIRT4 in the IPL did not change significantly, it became fractured and discontinuous. We observed that GS was downregulated in SIRT4^KO^ mouse retinas and that MGCs exhibited a disrupted radial morphology of MGCs (Figure 3F). The results indicated that SIRT4 deletion scarcely affected the retinal structure of mice, but downregulated the expression of GS protein.

### Retinal Electrophysiological Function of 2-Month Sirtuin 4-Deficient Mice

**FIGURE 4:**
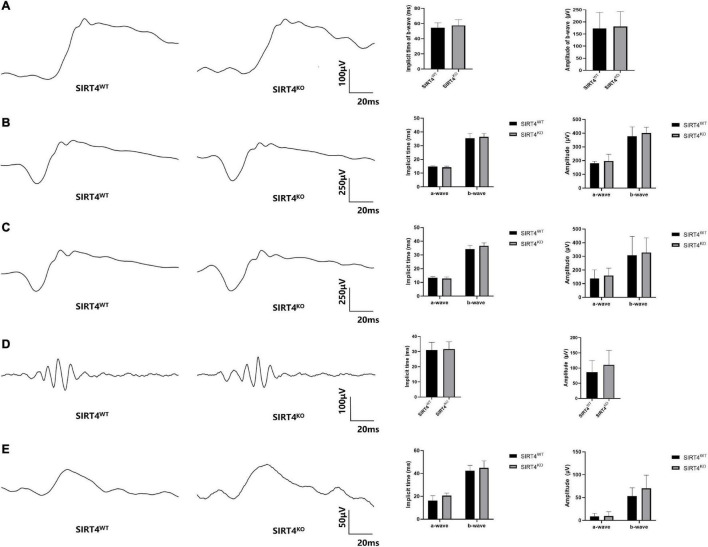
Electroretinogram of 2-month SIRT4-deficient mice. **(A)** Scotopic 0.01 ERG waveforms analysis of SIRT4^KO^ mice and SIRT4^WT^ mice. **(B)** Scotopic 3.0 ERG waveforms analysis of SIRT4^KO^ mice and SIRT4^WT^ mice. **(C)** Scotopic 10.0 ERG waveforms analysis of SIRT4^KO^ mice and SIRT4^WT^ mice. **(D)** Scotopic 3.0 oscillatory potential ERG waveforms analysis of SIRT4^KO^ mice and SIRT4^WT^ mice. **(E)** Photopic 3.0 ERG waveforms analysis of SIRT4^KO^ mice and SIRT4^WT^ mice. WT: wild type; KO: knock out. Data are shown as mean ± SEM. (*n* = 3 per group).

To explore the effect of SIRT4 deletion on retinal electrophysiology in mice, we conducted electroretinogram (ERG) in SIRT4^KO^ mice and SIRT4^WT^ mice. The ERG showed no significant difference in implicit time and amplitude between SIRT4^KO^ mice and SIRT4^WT^ mice in the scotopic 0.01, 3.0, and 10.0 categories (Figures 4A–C). In the scotopic 3.0 oscillatory potential, the implicit time was similar and the amplitude difference was not significant (Figure 4D). Although the ERG waveform in the photopic 3.0 was not completely consistent, there was no significant difference between the SIRT4^KO^ and SIRT4^WT^ groups (Figure 4E). The ERG results revealed that SIRT gene knockout had no significant effect on retinal electrophysiological function in mice.

### Resveratrol Upregulated SIRT4 and GS Protein Expression in Mice

**FIGURE 5:**
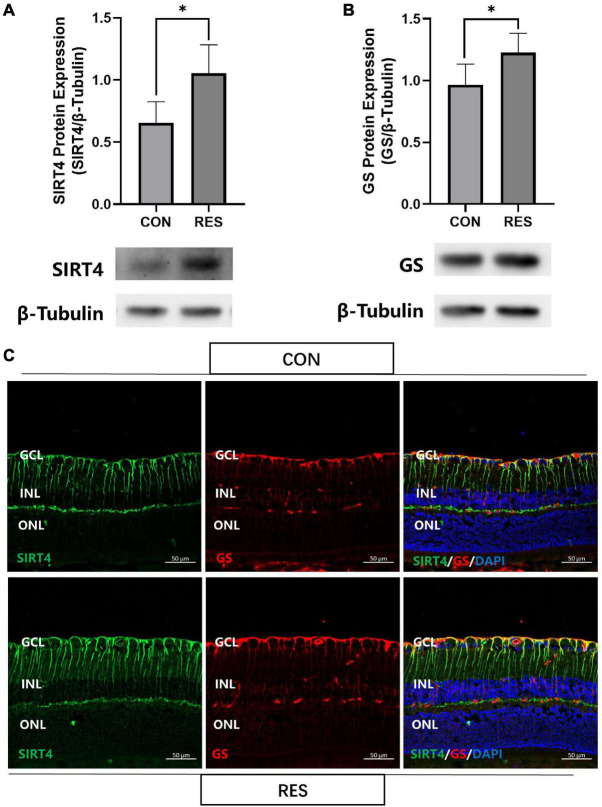
Resveratrol up-regulate SIRT4 and GS protein expression in 2 month mice retinas. Western blot analysis of SIRT4 **(A)** and GS **(B)** expression. **(C)** Immunofluorescence analysis of SIRT4 and GS in the retina of normal control group and RES intraperitoneal injection group mice. CON: normal control; RES: resveratrol intraperitoneal injection; GS: glutamine synthetase. Data are shown as mean ± SEM. (*n* = 5 per group, *P < 0.05).

We previously found that resveratrol significantly increased SIRT4 protein expression in zebrafish retina (Sheng et al., 2018). With this result in mind, we intraperitoneally injected 2-month mice with RES (20 mg/kg) for five consecutive days. SIRT4 (Figure 5A) and GS (Figure 5B) protein expression significantly increased following administration of RES. Filamentous staining of SIRT4 in the retina of RES intraperitoneal injection group was denser and longer than that of normal control group, so as the GS (Figure 5C).

### Resveratrol Protected Mice Retines From Excitatory Neurotoxic Damage

**FIGURE 6:**
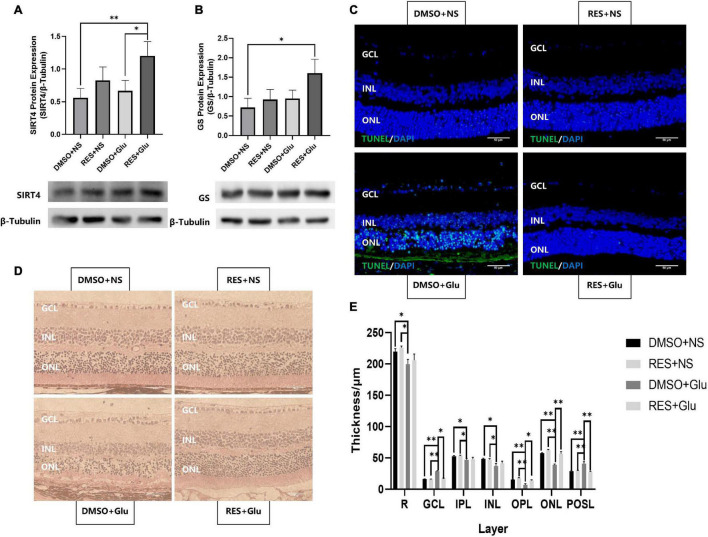
Resveratrol protected mice retines from excitatory neurotoxic damage. Western blot analysis of SIRT4 **(A)** and GS **(B)** protein expression of DMSO+NS, RES+NS, DMSO+Glu and RES+Glu. **(C)** TUNEL labeling of retinal section 3 day after glutamate injury showed that apoptosis was attenuated in RES+Glu compared to DMSO+Glu. **(D)** H&E staining of mice retina Paraffin section. **(E)** Retinal thickness analysis showed a significant reduction in retinal thickness of DMSO+Glu and there was no signifucant changes in that of RES+Glu. GCL, ganglion cell layer; IPL, inner plexiform layer; INL, inner nuclear layer; OPL, outer plexiform layer; ONL, outer nuclear layer; POSL, photoreceptor outer segment layer. GS, glutamine synthetase; DMSO, dimethylsulfoxide; RES, resveratrol; NS, normal saline; Glu, glutamate; DMSO+NS: DMSO (6.67%) was intraperitoneal injection every 2 days from postnatal 20 days to 2 months of age and Glu (50 nmol) was injected intravitreal 3 days before sampling; RES+NS: RES (20 mg/L) soluble in DMSO (6.67%) was intraperitoneal injection every 2 days from postnatal 20 days to 2 months of age and NS was injected intravitreal 3 days before sampling; DMSO+Glu: DMSO (6.67%) was intraperitoneal injection every 2 days from postnatal 20 days to 2 months of age and Glu (50 nmol) soluble in NS was injected intravitreal 3 days before sampling; RES+Glu: RES (20 mg/L) soluble in DMSO (6.67%) was intraperitoneal injection every 2 days from postnatal 20 days to 2 months of age and Glu (50 nmol) soluble in NS was injected intravitreal 3 days before sampling. TUNEL was used to stain apoptotic cell (green) and DAPI was used to stain the nuclei (blue). Data are shown as mean ± SEM. (*n* = 3 per group, *P < 0.05, ^**^P < 0.01). Scale bar = 50 μm.

Previous studies have shown that GS can regulate retinal glutamate metabolism, and reduce apoptosis (Bui et al., 2010). As RES upregulates the expression of SIRT4 and GS, we hypothesized that RES could protect mice retines from excitatory neurotoxic damage. Thus, we injected large doses of glutamate (50 nmol) intravitreally into mice and analyzed the effects of long-term administration of RES on glutamate injury. The eyeball was removed 3 days after the injury. The results showed that RES upregulated SIRT4 and GS protein expression in the glutamate injury group (Figures 6A,B). TUNEL staining showed that intravitreal injection of glutamate caused retinal GCL, INL and ONL cell apoptosis similar to kainate (a kind of glutamate receptor agonist) (Fleming et al., 2019), while there was no significant cell apoptosis in the group with RES (Figure 6C). After glutamate injury, the retinal structure of all layers was obviously disordered, the GCL became edematous, the nuclei of the GCL layer and ONL layer were scattered, and the photoreceptor outer segment layer (POSL) was broken (Figure 6D). In addition, scattered cells are seen in the vitreous cavity. The retinal thickness analysis showed a significant reduction in the thickness of the retina, the OPL and the ONL, and incrassation of the GCL thickness after glutamate injury while they were similar to the control with the help of RES (Figure 6E).

## Discussion

Our study revealed for the first time that the role of SIRT4 in the retina is highly correlated with MGCs. We found that SIRT4 was co-expressed with MGCs markers (GS and Vimentin). The expression of SIRT4 protein in the retina increased with postnatal time and reached its peak at 2 months with diffuse changes in its expression pattern. The distribution of SIRT4 protein was similar to that of MGC markers (GS and CRALBP) during the development of MGCs (Bachleda et al., 2016). SIRT4 gene knockout did not affect the structure and electrophysiological function of mouse retina under basal conditions but resulted in the downregulation of GS protein expression. In addition, our experiments suggested that RES, as a SIRT4 activator, can mitigate excitatory neurotoxic damage caused by glutamate excess.

Previous studies have shown that SIRT4 is highly expressed in glial cells, specifically astrocytes, in the brain and radial glia (Komlos et al., 2013). SIRT4 was filamentously localized in cells that were likely radial glia and co-expressed with vimentin and nestin. Similar expression patterns of SIRT4 were observed in our experiments. GS and vimentin, as specific markers of MGCs (Hayashi et al., 2021), have similar protein expression and fluorescence localization to SIRT4, suggesting that SIRT4 may play a role in MGCs. Given that MGCs play a crucial role in retinal function and structure, we were curious about the role of SIRT4 in the retina. Therefore, we further analyzed the effects of SIRT4 gene deletion on the structure and function of MGCs and the retina. The loss of SIRT4 had no significant effect on retinal structure or electrophysiological function under basal conditions, which was consistent with the results of Lin et al. (2018). Three reasons may explained this situation. First, SIRT4 had a low expression level in the early stage of the retina and had little influence on retinal development. Then, the phenotype of SIRT family knockout mice was insidious. The structure and function of mouse retina may not change under basic conditions, but it tends to change under specific stimuli like SIRT3 and SIRT5 (Lin et al., 2016). Finally, the function of SIRT4 may compensate for other sirtuins, such as SIRT1 and SIRT3, which have been confirmed to be increased in SIRT4^KO^ mice (Nasrin et al., 2010; Balaiya et al., 2017). We speculate that the increase of other sirtuins in SIRT4 KO mice may partially compensate for the function of SIRT4, and the increase in SIRT1 protein was found in our preliminary. We found that the expression of GS was downregulated and discontinuous in SIRT4-deficient mice. After upregulating SIRT4 expression by RES, GS showed enhanced radial filamentous expression and increased protein expression consistent with SIRT4. These results convinced us that SIRT4 may be involved in the regulation of GS expression in MGCs.

Sirtuin 4 inhibits the enzymatic activity of glutamate dehydrogenase (GDH) and limits the metabolism of glutamate and glutamine to produce ATP (Haigis et al., 2006; Fernandez-Marcos and Serrano, 2013). GDH and SIRT4 play opposing roles in the development of astroglia from radial glia in the central nervous system (Komlos et al., 2013). Interestingly, we observed that SIRT4 and GS may play similar roles in the development of MGCs. Glutamine immunoreactivity was highest in horizontal cells and MGC endfeet (Bui et al., 2010), which corresponded to the localization of SIRT4 in the retina, indicated that SIRT4 plays a role in regulating glutamate-glutamine metabolism in the retina. By excess glutamate intravitreal injection, we found that intraperitoneal injection of RES in mice upregulated the expression of SIRT4 and GS and reduced the apoptosis of retinal cells induced by glutamate, indicating the regulatory effect of SIRT4 on glutamate through GS and its protective effect on the retina. Inhibition of Müller glial cell glutamine synthetase blocked glutamatergic neurotransmission, which could contribute to neuronal degeneration and the animals became functionally blind (Barnett et al., 2000; Bringmann et al., 2013). Brain studies in SIRT4 knockout mice have also shown that SIRT4 has a neuroprotective effect against excitatory toxic injury by promoting GLT-1-dependent glutamate uptake (Shih et al., 2014). Combined with this study, we hypothesized that SIRT4 may regulate the retinal glutamate-glutamine cycle through multiple pathways. Considering that excitotoxicity caused by glutamate is an important part of glaucoma injury, investigating the role of SIRT4 in glutamate-glutamine metabolism is of great significance.

Resveratrol, a natural polyphenol compound found chiefly in grapes and wine, is regarded as an antioxidant, anti-inflammatory agent, anti-apoptotic agent and antineoplastic agent (Malhotra et al., 2015). It has been reported to inhibit oxidative stress in diabetic cells, mitigate the effects of retinal ischemic injury in rats, and inhibit pathologic retinal neovascularization in very low-density lipoprotein receptor mutant mice (Hua et al., 2011; Luo et al., 2018; Popescu et al., 2018). Human sirtuin isoforms are considered attractive therapeutic targets for neurodegenerative disorders (Dai et al., 2018). Activation of SIRT4 expression by RES may have a protective effect on neurodegenerative diseases, which was preliminarily validated in mice in our study. In addition, we found that resveratrol inhibits the Akt/mTOR pathway in the retina (not shown). Previous studies have demonstrated that resveratrol can inhibit mTOR signaling induced autophagy, which can mediate many beneficial effects, including protection against oxidative stress damage (Park et al., 2016). Experiments on zebrafish and retinal pigment epithelial cells also showed that resveratrol could downregulate the Akt/mTOR pathway, promote autophagy and improve mitochondrial function (Wang et al., 2019; Josifovska et al., 2020). The role of SIRT4 in the mechanisms above needs to be further studied.

In summary, we found co-localization of SIRT4 and MGCs, and SIRT4 was involved in the role of MGCs and MGC maker GS. The results above provide a direction for the follow-up study of retinal SIRT4. However, this study did not further study SIRT4 gene knockout mice under stress state, nor did it discuss the role of other SIRT family proteins. In addition, we also observed that SIRT4 was highly expressed in choroid and ciliary body epithelium in mice, and its significance remains to be further studied.

## Conclusion

We show for the first time that SIRT4 co-localized with Müller glial cell markers (including vimentin and glutamine synthetase) in human, mouse and rat retinas. Resveratrol, as a SIRT4 activator, increased the expression of glutamine synthetase protein and protected the mice retina against excitotoxicity caused by excessive glutamate. Upregulation of SIRT4 expression may be a new direction for the treatment of retinal neuronal degeneration caused by glaucoma and age-related macular degeneration.

## Data Availability Statement

The original contributions presented in the study are included in the article/supplementary material, further inquiries can be directed to the corresponding author/s.

## Ethics Statement

The studies involving human participants were reviewed and approved by Affiliated Eye Hospital of Nanchang University. Written informed consent to participate in this study was provided by the participants’ legal guardian/next of kin. The animal study was reviewed and approved by Affiliated Eye Hospital of Nanchang University. Written informed consent was obtained from the owners for the participation of their animals in this study.

## Author Contributions

WW, PH, and XZ conceived the study. WW, PH, MQ, SY, and ZX performed the experiments. WW, FW, GC, MJ, and XZ analyzed the data. WW, PH, and XZ drafted the manuscript. All authors read and approved the final version of manuscript.

## Conflict of Interest

The authors declare that the research was conducted in the absence of any commercial or financial relationships that could be construed as a potential conflict of interest.

## Publisher’s Note

All claims expressed in this article are solely those of the authors and do not necessarily represent those of their affiliated organizations, or those of the publisher, the editors and the reviewers. Any product that may be evaluated in this article, or claim that may be made by its manufacturer, is not guaranteed or endorsed by the publisher.
